# Air Plasma-Activated Medium Evokes a Death-Associated Perinuclear Mitochondrial Clustering

**DOI:** 10.3390/ijms23031124

**Published:** 2022-01-20

**Authors:** Manami Suzuki-Karasaki, Takashi Ando, Yushi Ochiai, Kenta Kawahara, Miki Suzuki-Karasaki, Hideki Nakayama, Yoshihiro Suzuki-Karasaki

**Affiliations:** 1 Department of Research and Development, Plasma ChemiBio Laboratory, Nasushiobara 329-2813, Japan; ksmanami181@gmail.com (M.S.-K.); ottyo.1308@gmail.com (Y.O.); smiki8613@gmail.com (M.S.-K.); 2 Department of Oral and Maxillofacial Surgery, Faculty of Life Sciences, Kumamoto University, Kumamoto 860-8556, Japan; k.k.ronbun@gmail.com (K.K.); hinakaya@kumamoto-u.ac.jp (H.N.); 3 Department of Orthopaedic Surgery, Yamanashi University School of Medicine, Yamanashi 409-3898, Japan; andou@yamanashi.ac.jp

**Keywords:** cancer, ROS, perinuclear mitochondrial clustering, mitochondrial network, mitochondrial positioning, plasma-activated medium, cell death, lipid peroxidation

## Abstract

Intractable cancers such as osteosarcoma (OS) and oral cancer (OC) are highly refractory, recurrent, and metastatic once developed, and their prognosis is still disappointing. Tumor-targeted therapy, which eliminates cancers effectively and safely, is the current clinical choice. Since aggressive tumors are substantially resistant to multidisciplinary therapies that target apoptosis, tumor-specific activation of another cell death modality is a promising avenue for meeting this goal. Here, we report that a cold atmospheric air plasma-activated medium (APAM) can kill OS and OC by causing a unique mitochondrial clustering. This event was named monopolar perinuclear mitochondrial clustering (MPMC) based on its characteristic unipolar mitochondrial perinuclear accumulation. The APAM caused apoptotic and nonapoptotic cell death. The APAM increased mitochondrial ROS (mROS) and cell death, and the antioxidants such as *N*-acetylcysteine (NAC) prevented them. MPMC occurred following mitochondrial fragmentation, which coincided with nuclear damages. MPMC was accompanied by mitochondrial lipid peroxide (mLPO) accumulation and prevented by NAC, Ferrostatin-1, and Nocodazole. In contrast, the APAM induced minimal cell death, mROS generation, mLPO accumulation, and MPMC in fibroblasts. These results suggest that MPMC occurs in a tumor-specific manner via mitochondrial oxidative stress and microtubule-driven mitochondrial motility. MPMC induction might serve as a promising target for exerting tumor-specific cytotoxicity.

## 1. Introduction

Intractable cancers such as osteosarcoma (OS) and oral cancer (OC) are greatly refractory, recurrent, and metastatic once developed, and their prognosis is still disappointing. Tumor-targeted therapy, which eliminates cancers effectively and safely, is the current clinical choice. Activating tumor-selective cell death is a promising approach for meeting this goal. Apoptosis is the primary modality of cancer cell death caused by diverse chemical and physical stresses as well as multidisciplinary therapies, including anticancer agents. However, it is evident that these aggressive tumors have cellular machinery protecting them from apoptosis, conferring inherent or acquired resistance [1,2,3]. An emerging view is that apoptosis is not the sole mode of cancer cell death. Several different means of cell death, such as autophagy and necroptosis, also occur in response to anticancer agents [4,5]. Thus, the activation of nonapoptotic cell death can be a promising alternative approach to treating intractable cancers.

Cold atmospheric plasma (CAP) has recently emerged as a promising tumor-targeting approach. Direct CAP treatment inhibits cell proliferation, migration, and invasion. It triggers different cell death modalities, including apoptosis, necrosis, and autophagy in vitro in various cancer cell lines as well as primary cancerous cells and tissues [6,7,8,9,10]. CAP also reduced the cell growth of xenografted tumors in vivo while exhibiting minimal toxicity in normal cells and tissues [7,9]. These properties have attracted much attention in cancer treatment. Exposing CAP to various solutions results in plasma-activated liquids (PALs), such as plasma-activated media (PAM). PALs have different chemical and biological effects depending on the physical properties of the plasma generated and various experimental parameters. Despite such variation, different types of PAM have antitumor activity against tumor cells both in vitro and in vivo while sparing normal cells and tissues [11,12,13,14,15,16]. Helium plasma-activated media (HePAM) and plasma-activated infusion (PAI) activate autophagy and necroptosis in a tumor-selective manner [13,14,17]. These PALs also have antitumor activity against OS in vitro and in vivo with minimal side effects. They can be easily administered systematically or locally to deep tissues by infusion and endoscope. Thus, they will promise a potent and safe approach for cancer treatment.

An accumulating body of evidence suggests that reactive oxygen and nitrogen species (ROS/RNS) play a vital role in mediating the antitumor activity of PALs. They commonly contain long-lived oxidants in the order of μM to mM, including hydrogen peroxide (H_2_O_2_), nitrite (NO_2_^−^), and nitrate (NO_3_^−^). H_2_O_2_ plays a significant role in PAM cytotoxicity [11,12,13,14,17] and tumor-targeting activity [13]. Notably, numerous reports showed synergistic effects between H_2_O_2_ and NO_2_^−^, although the molecular targets of the synergism are poorly understood [18,19,20,21].

Mitochondria are highly dynamic organelles, constantly changing their size, shape, and location to cope with the energy demands [18,19]. A growing body of evidence indicates that mitochondrial network dynamics play a critical role in regulating functions and survival in non-transformed and transformed cells. The dynamics are regulated by the delicate balance between fission and fusion of the mitochondrial membrane. Dynamin-related proteins (Drps) with GTPase activity such as Drp1, optic atrophy 1 (OPA1), and mitofusin 1/2 (Mfn1/2) act in concert to regulate the dynamics; Drp1 regulates fission, while OPA1 and Mfn1/2 control fusion and cristae organization. A defect in either process causes severe mitochondrial and cellular dysfunctions [22,23]. Mitochondrial fission facilitates the elimination of damaged mitochondria through mitophagy-mediated disruption. Therefore, its deficiency leads to a hyperfused mitochondrial network and compromised mitochondrial quality control [24]. Conversely, mitochondrial fusion facilitates the exchange of mitochondrial DNA and the metabolites required for mitochondrial function. Accordingly, its failure leads to mitochondrial fragmentation, reduced mitochondrial DNA, growth, mitochondrial membrane potential (ΔΨ_m_), and defective respiration [25]. Mitochondrial motility and subcellular positioning have recently emerged as another critical regulatory factor in mitochondrial and cellular functions [26,27]. Mitochondria are clustered around perinuclear sites in the perinuclear mitochondrial clustering (PNMC). PNMC has been suggested to maintain the hypoxic status of cancer cells via mitochondrial ROS production and stabilization of the hypoxia-inducible factor 1α (HIF-1α), the master transcription factor regulating hypoxic signaling [28,29,30,31].

We have previously reported that the tumor necrosis factor-related apoptosis-inducing ligand (TRAIL) induces mitochondrial fragmentation by increasing Drp1 phosphorylation at Ser 616. The Drp1 inhibitor Mdivi-1 and Drp1 gene silencing augment TRAIL-induced caspase-3 activation, mitochondrial depolarization, and apoptosis [32,33]. These facts indicate that the Drp1-dependent mitochondrial fragmentation is cytoprotective. The enhanced apoptosis pathways are associated with the shift from mitochondrial fragmentation to clustering, and plasma membrane depolarization causes it [33]. Strikingly, such a shift occurs in OS, malignant melanoma, and lung cancer but not in normal cells. HePAM [12,13] and PAI [17] show similar effects in various cancer cells. They quickly cause mitochondrial fragmentation at low (subtoxic) concentrations while causing mitochondrial clustering at high (toxic) doses. Alternatively, even when applied at high concentrations, they caused substantial fragmentation only in noncancerous cells, such as melanocytes and fibroblasts. Together, these facts suggest that cancers are more prone than normal cells to mitochondrial clustering, and this higher susceptibility may lead to tumor-specific cell death induction. Thus, it is worth knowing whether the given antitumor agent can evoke such mitochondrial clustering.

Different gases such as Argon, Helium, Nitrogen, or a mixture of gases can produce CAP. Many CAP experiments utilize inert gases, such as Argon and Helium, as a CAP source because they are easier to ionize than ambient air. Recently, we noticed that air bubbling augmented the antitumor activity of HePAM, suggesting the involvement of air components in action. Therefore, we used ambient air instead of helium as a CAP source to enrich the active substances, generating an air plasma-activated medium (APAM). The APAM was more effective than HePAM and PAI in reducing cell growth in multiple cancers.

In the present study, we explored the antitumor activity of the APAM with a particular interest in its impact on the mitochondrial network and positioning. We found that the APAM induced nonapoptotic cell death via a unique death-associated mitochondrial perinuclear clustering in a tumor-specific manner.

## 2. Results

### 2.1. APAM Has Potent Antitumor Activity against OS In Vitro and In Vivo

**Figure 1:**
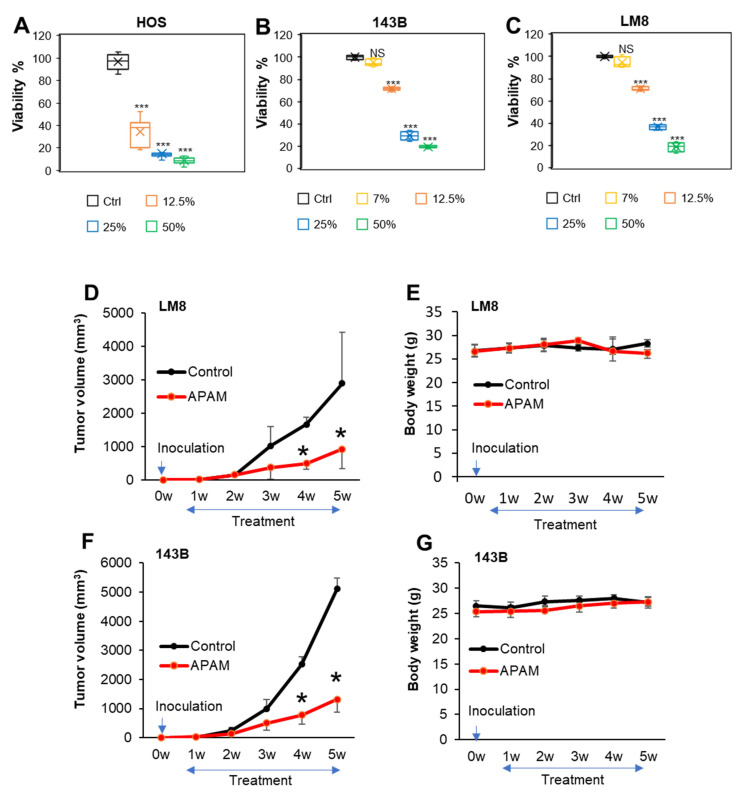
APAM has potent antitumor activity against OS in vitro and in vivo. (**A**–**C**). HOS (**A**), 143B (**B**), and LM8 (**C**) cells (4 × 10^3^ cells) were treated with the indicated concentrations of the APAM for 72 h and analyzed for viability using a WST-8 cell growth assay. Data are the mean ± SD (*n* = 4–9). Data were analyzed by one-way analysis of variance followed by Tukey’s post hoc test. *** *p* < 0.001; NS, not significant vs. control treated with vehicle. (**D**–**G**). C3H (**D**,**E**) and nude mice (**F**,**G**) were inoculated with 1 × 10^6^ each of LM8 (**D**,**E**) and 143B cells (**F**,**G**), respectively at day 0, and intravenously administered 200 μL of APAM (50%) 3 times per week for 4 weeks from day 7. The sizes of the tumors in the mice (**D**,**F**) and mice’ weights (**E**,**G**) were measured weekly. Values represent the mean ± SD (*n* = 6). * *p* < 0.05 vs. the control treated with vehicle.

First, we examined whether the APAM had antitumor activity in vitro. Cells were treated with the APAM at different concentrations (7, 12.5, 25, 50% solution) for 72 h and analyzed for cell viability by a WST assay. The APAM treatment significantly reduced cell viability in HOS, LM8, and 143B OS cells in a dose-dependent manner (Figure 1A–C). However, the degree of the reduction varied considerably depending on the cell line. The APAM (≤12.5%) caused a substantial effect (≥50% reduction) in high responders, including human HOS cells. In comparison, the APAM (≥25%) was necessary for a ≥50% reduction in low responders, such as human 143B and murine LM8 cells (Figure 1A–C). The effect was preceded by cellular and nuclear morphological changes, including appearing in blebbed, nonadherent, round cells with shrunken or fragmented nuclei. These changes were observed within 2 h after the APAM addition. Next, we examined whether the APAM had antitumor activity in vivo. The LM8 cells can be transplanted into the host C3H mice to develop solid tumors. We assessed the action in the allograft model under the regimen shown in Supplementary Figure S1. Subcutaneous inoculation of the cells into mice resulted in rapid tumor growth, reaching about 3000 mm^3^ within five weeks. Intravenous administration of the APAM (50%) three times a week resulted in a significant (68.1%) reduction in the tumor size (Figure 1D). The APAM was also effective in a xenograft model where human 143B cells were inoculated into nude mice. The tumor developed about 5000 mm^3^ within five weeks after inoculation. There was a minimal difference in mice’s weights between the treated and untreated groups (Figure 1E,G). The APAM administration resulted in a significant (74.2%) reduction in the tumor size (Figure 1F). Also, no weakness, diarrhea, abnormal coat, anorexia, or abnormal behavior were observed throughout the experiments. Thus, the APAM slowed tumors’ initiation and reduced the growth rate with minimal side effects. These results indicate that the APAM has potent antitumor activity against OS in vitro and in vivo.

### 2.2. APAM Can Activate Apoptosis and Nonapoptotic Cell Death in OS Cells

**Figure 2:**
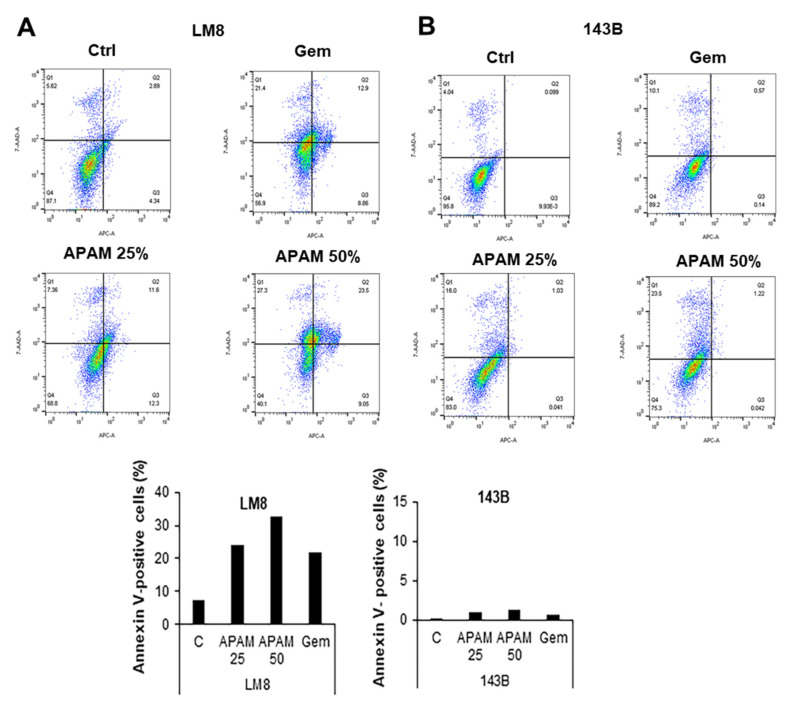

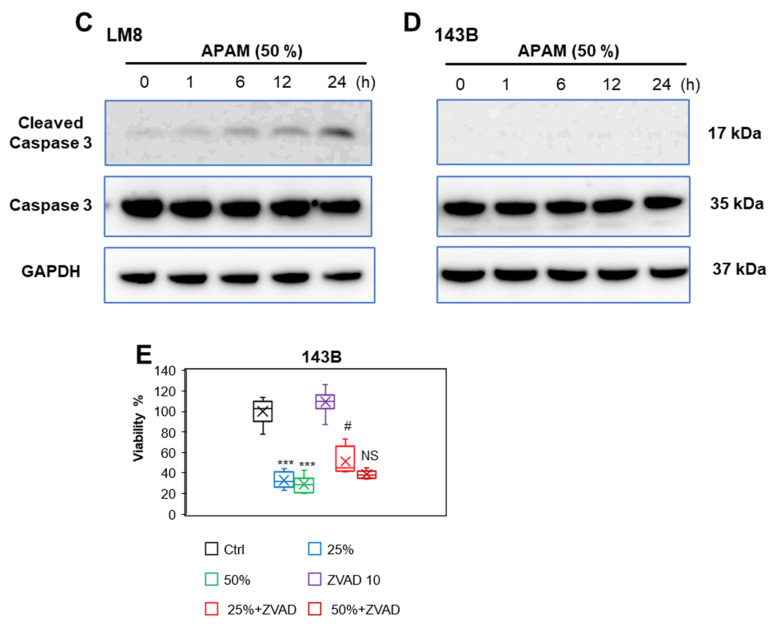
APAM induces apoptotic and nonapoptotic cell death. (**A**,**B**). LM8 (**A**) and 143B (**B**) cells were treated with the APAM (25, 50%) for 24 h, stained with Annexin V-APC and 7-AAD, and analyzed by flow cytometry. Gemcitabine (Gem) was used as a positive control for apoptosis induction. The ratios of apoptotic (Annexin-V-positive) cells were shown in the right panels. (**C**,**D**). LM8 (**C**) and 143B cells (**D**) were treated with the APAM (50%) for the indicated time and then analyzed for the expression of the full-length and cleaved (active) Caspase-3 by Western blotting analysis using specific antibodies. GAPDH was used as the loading control. See Supplementary Figure S2 for examples of uncropped images and quantification for each antibody. (**E**). 143B cells were pretreated with 10 μM *Z*-VAD-FMK for 1 h and then treated with the APAM (25, 50%) for 72 h and analyzed for viability using the WST-8 cell growth assay. (**E**) APAM induces apoptotic and nonapoptotic cell death. Data are the mean ± SD (*n* = 4−9). Data were analyzed by one-way analysis of variance followed by Tukey’s post hoc test. *** *p* < 0.001 vs. control treated with vehicle, # *p* < 0.05; NS vs. APAM alone.

To investigate cell death modality, we analyzed Allophycocyanin (APC)-conjugated Annexin V and 7-aminoactinomycin D (7-AAD) double staining. The cells were treated with the APAM (25, 50% solution) for 24 h and analyzed for their Annexin V and 7-AAD double staining in a flow cytometer. Figure 2 shows the results obtained in LM8 and 143B cells. Gemcitabine (Gem) was used as an apoptosis inducer in OS [17]. The APAM resulted in dose-dependent increases in apoptotic (Annexin V-positive) cells and necrotic (Annexin V-negative) cells in LM8 cells (Figure 2A). Apoptosis was increased up to a maximum of 30% in response to the APAM (50%). Alternatively, the APAM caused minimal apoptotic cells while increasing necrotic cells in 143B cells in a dose-dependent manner (Figure 2B). Western blotting analyses using cleaved caspase-3-specific antibodies confirmed the flow cytometric observations. The APAM treatment increased the cleaved form of caspase-3 (17 kDa), a hallmark of apoptosis, over time in LM8 cells (Figure 2C) but not in 143B cells (Figure 2D). Additionally, the broad-spectrum caspase inhibitor Z-VAD-FMK could not entirely inhibit the APAM’s (50%) cytotoxic activity in 143B cells (Figure 2E). Conversely, the inhibitor attenuated the effect of the APAM (25%). These results indicate that the APAM can activate apoptosis or nonapoptotic cell death depending on the cell line and concentration.

### 2.3. ROS Plays a Vital Role in Cell Death

**Figure 3:**
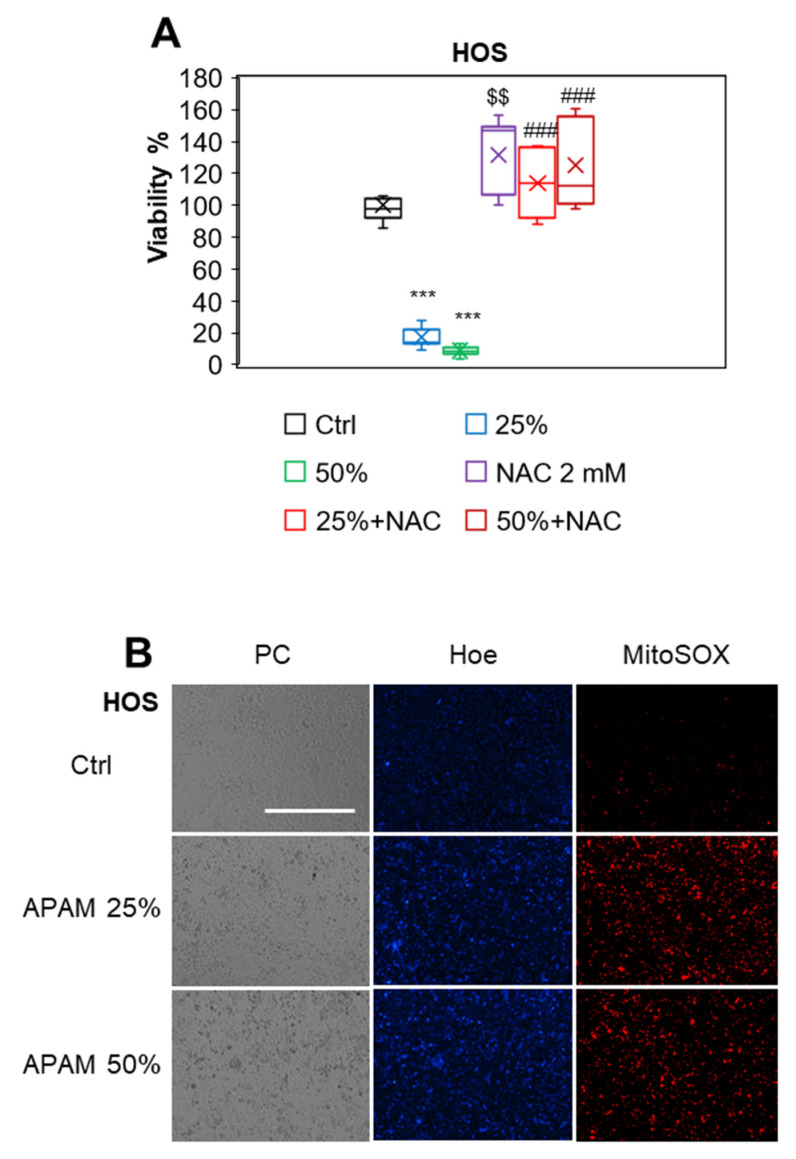
APAM induces cell death in a ROS-dependent manner. (**A**). HOS cells were treated with the APAM (25, 50%) or 2 mM NAC alone or in combination for 72 h and analyzed for viability using the WST-8 cell growth assay. Data are the mean ± SD (*n* = 8 or 9). Data were analyzed by one-way analysis of variance followed by Tukey’s post hoc test. *** *p* < 0.001; $$ *p* < 0.01 vs. control treated with vehicle, ### *p* < 0.001 vs. APAM alone. (**B**). HOS cells (1.5 × 10^4^) were treated with the APAM (25, 50%) for 2 h and then stained with 5 μM MitoSOX. The nuclei were counterstained with Hoechst 33342 (Hoe). Images were obtained with the EVOS FL Cell Imaging System equipped with a 10 × objective and analyzed using the freely available NIH ImageJ software. PC, phase contrast. Bar = 400 μm.

H_2_O_2_ is found in various PALs, including PAM, and represents the primary mediator of anticancer activity [11,12,13,14]. Therefore, we examined whether the APAM contained H_2_O_2_ by directly quantitating its contents within the APAM using Amplex Red. We found that the APAM contained <10 μM of H_2_O_2_ (6.1 ± 2.5 μM, *n* = 4) under the standard experimental conditions (1 min-exposure/mL medium). Next, we determined whether ROS contributed to the APAM’s cytotoxic activity. Cells were pretreated with the antioxidant *N*-acetylcysteine (NAC) for 1 h and then treated with the APAM (25, 50%) for 72 h. The NAC treatment entirely prevented the decrease in cell viability in HOS cells (Figure 3A), suggesting the involvement of ROS in cell death. To further explore the role of ROS, we examined the APAM’s ability to induce intracellular ROS production. The APAM significantly increased mitochondrial superoxide in the cells, as shown by the mitochondria-localizing superoxide-reactive probe MitoSOX Red (MitoSOX) (Figure 3B). These results indicate that ROS plays a vital role in OS cell death caused by the APAM.

### 2.4. APAM Evokes MPMC in a Tumor-Specific Manner

**Figure 4:**
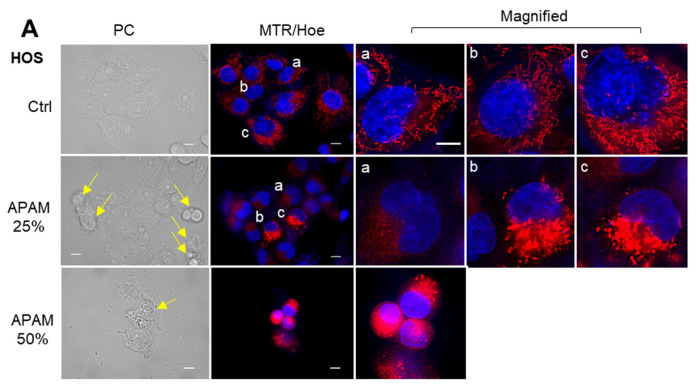

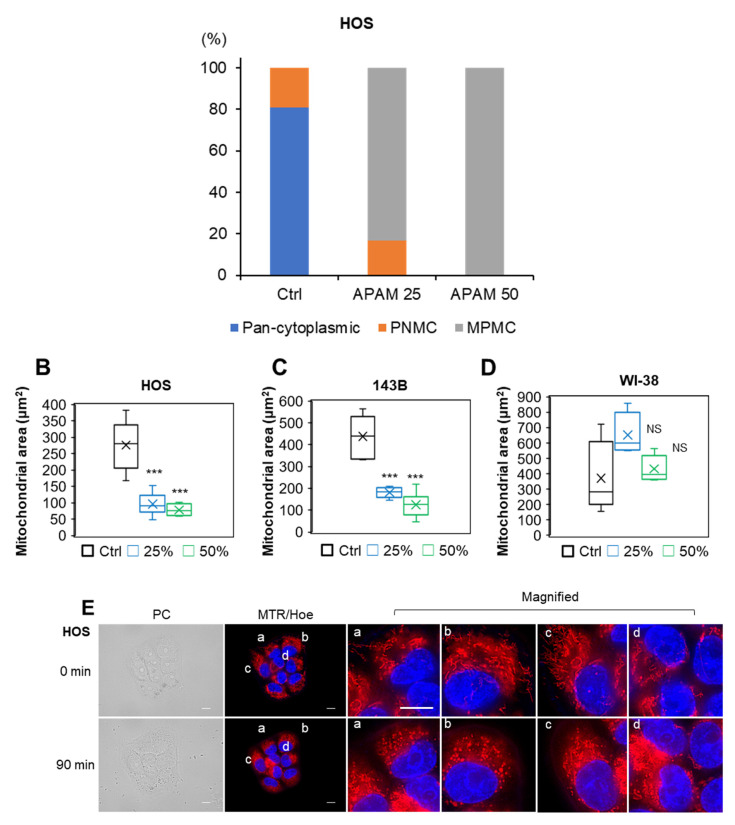

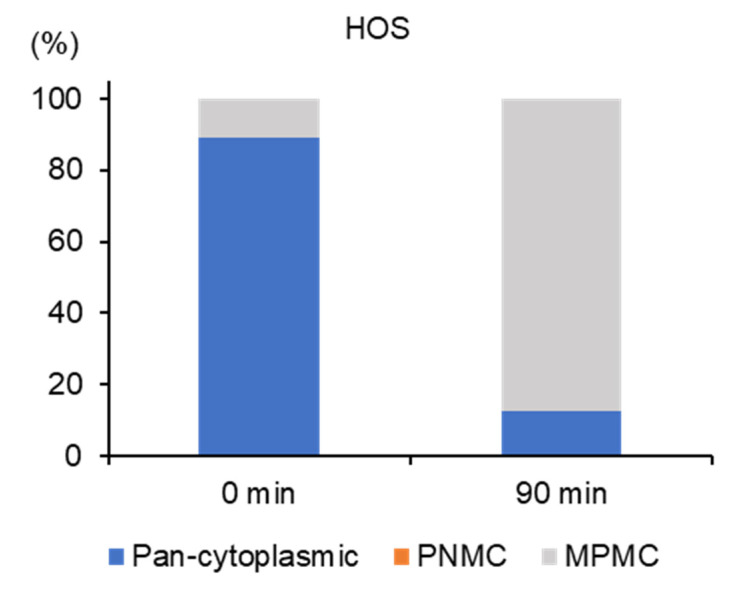
APAM induces MPMC in a tumor-specific manner. (**A**,**E**). After treating HOS cells with the APAM (25, 50%) for 18 h (**A**), 0 (no treatment), or 90 min (**E**), mitochondria and nuclei were stained with MTR and Hoechst 33342, respectively. Images were obtained using a BZ X-710 Digital Biological Microscope equipped with a 100×, 1.40 n.a. UPlanSApo Super-Apochromat coverslip-corrected oil objective, and analyzed using BZ-H3A application software. Magnified panels (a–d) show the magnification of a–c in the MTR/Hoe merged images. In (**A**), the yellow arrows indicate damaged or dying cells. Bar = 10 μm. For each experimental group, cells (≤40) in at least two different pictures were counted for three different subcellular distributions (pan-cytoplasmic, PNMC, and MPMC), and the percentage was shown (*n* = 2–4). (**B**–**D**). HOS (**B**), 143B (**C**), and WI-38 cells (**D**) were treated with the APAM (25, 50%) for 18 h, and mitochondria and nuclei were stained with MTR and Hoechst 33342 (Hoe), respectively. Images were taken as described above. Then, the occupied mitochondrial area in three different images per sample was measured using the BZ-H3A application, as shown in Supplementary Figure S4. Data are the mean ± SD (*n* = 4–9). Data were analyzed by one-way analysis of variance followed by Tukey’s post hoc test. *** *p* < 0.001; NS, not significant vs. control treated with vehicle. (**E**) HOS cells were treated with the APAM (50%) for 90 min, and mitochondria and nuclei were stained with MTR and Hoechst 33342 (Hoe), respectively. Images were taken as described above. Magnified panels (a–d) show the magnification of a–d in the MTR/Hoe merged images. Mitochondria with the three distributions were counted as described above.

Next, we determined whether the APAM affected the mitochondrial network in OS cells. After the APAM treatment, the mitochondria in live cells were stained with MitoTracker Red (MTR), and the nuclei were counterstained with Hoechst 33342. The mitochondria in the untreated HOS cells had substantial membrane potential and exhibited a reticular network (Figure 4A, upper panels). Following the APAM treatment, the mitochondria became fragmented and clustered concomitantly with altered membrane potential, as indicated by the potential-dependent MTR signals (Figure 4A, lower panels). The mitochondrial abnormalities, including the membrane depolarization, were explicitly seen in the round damaged cells (indicated by yellow arrows), indicating that they are death-associated events. Moreover, we noticed that the mitochondrial network collapse was associated with altered mitochondrial subcellular positioning. In untreated cells, the mitochondria are widely distributed through the cytoplasm (pan-cytoplasmic) or all-around or on both sides of the perinuclear sites (PNMC) (Figure 4A, upper panels). Alternatively, following the APAM treatment, most mitochondria clustered at one side of the perinuclear sites (Figure 4A, lower panels). Similar altered mitochondrial morphology and positioning were seen in 143B cells (Supplementary Figure S4). The modified positioning was associated with massive nuclear damages. A quantitative measurement of the mitochondria-occupied area revealed a significant reduction in APAM-treated HOS and 143B cells compared with untreated cells (Figure 4B,C). Conversely, no significant decrease was seen in fibroblasts (Figure 4D). We found that such alterations in mitochondrial morphology and distribution became pronounced as rapidly as within 2 h after adding APAM cells. Figure 2E shows representative results in HOS cells. The mitochondria became fragmented and accumulated in the perinuclear regions at 90 min after the APAM treatment, although minimal cell morphological changes, such as loss of membrane integrity, were seen at the timepoint. These results indicate that the APAM modulates mitochondrial positioning in a tumor-specific manner and that this event may cause cell death. Therefore, we named this event monopolar perinuclear mitochondrial clustering (MPMC) and investigated the mechanisms underlying it in more detail.

### 2.5. MPMC Occurs in a Microtubule- and ROS-Dependent Manner

**Figure 5:**
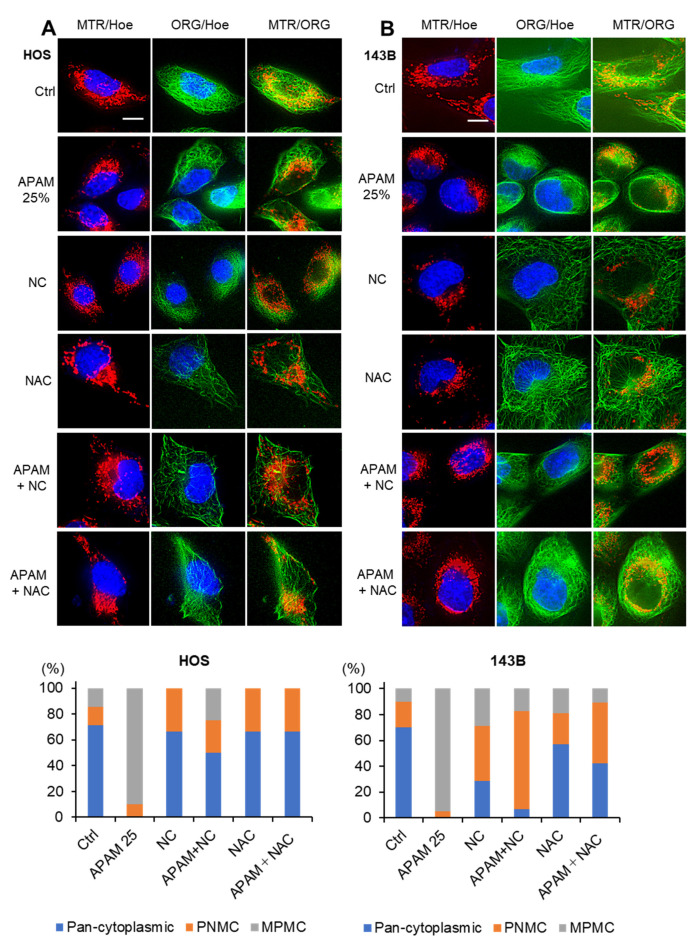
MPMC results from a microtubule- and ROS-dependent mitochondrial motility. HOS (**A**) and 143B cells (**B**) were pretreated with 100 nM Nocodazole (NC) or 2 mM *N*-acetylcysteine (NAC) for 1 h and then treated with the APAM (25%) for 2 h. Mitochondria, tubulin, and nuclei were stained with MTR, Oregon Green Paclitaxel (ORG), and Hoechst 33342 (Hoe), respectively. Images were taken as described in the legend of Figure 4. Bar = 10 μm. For each experimental group, mitochondria showing the three distributions were counted as described in the legend of Figure 4.

Mitochondria can move throughout the cytoplasm through the action of two microtubule-associated motor proteins, Kinesin and Dynein [34,35,36,37]. To determine the role of microtubules in MPMC, we attempted to analyze the tubulin and mitochondrial dynamics simultaneously. After the APAM treatment, the mitochondria and tubulin in HOS cells were stained with MTR and Oregon Green Paclitaxel, respectively, and observed microscopically. Similar to the mitochondria, the tubulin exhibited a network distributing broadly throughout the cytoplasm in untreated cells (Figure 5A, top panels). Following the APAM treatment, the tubulin network was mainly distributed to one side of the nuclei (Figure 5A, second panels). Treatment with the microtubule inhibitor Nocodazole (NC) or the antioxidant NAC considerably abolished the tubulin network with minimal effects on the tubulin’s subcellular distribution (Figure 5A, third and fourth panels). Notably, NC and NAC blocked MPMC and tubulin redistribution (Figure 5A, fifth and bottom panels). Similar results were obtained in 143B cells (Figure 5B). These results indicate that MPMC occurs in a microtubule- and ROS-dependent manner.

### 2.6. MPMC Involves LPO Accumulation and Cardiolipin (CL) Oxidation in the Mitochondrial Cluster

**Figure 6:**
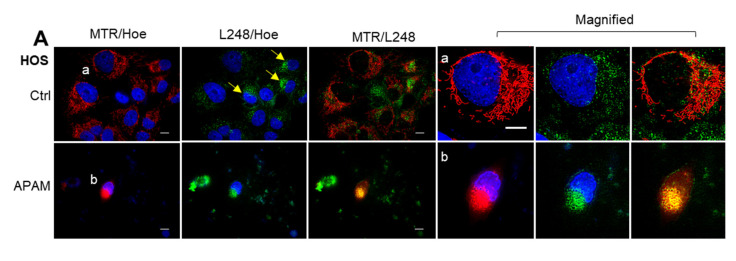

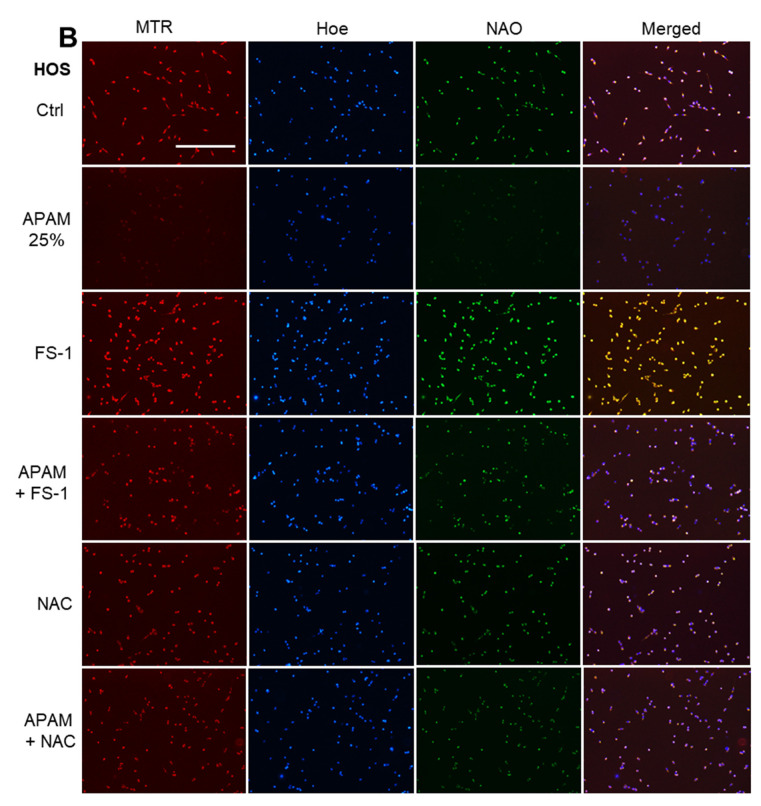
MPMC involves lipid peroxides (LPO) accumulation and Cardiolipin (CL) oxidation. (**A**). HOS cells were treated with the APAM (50%) for 2 h, and mitochondria, LPO, and nuclei were stained with MTR, L248, and Hoechst 33342 (Hoe), respectively. Images were taken as described in the legend of Figure 4. The magnified images (a,b) show the magnification of a,b in the merged images. Bar = 10 μm. (**B**). HOS cells were pretreated with 1 μM Ferrostatin-1 (FS-1) or 2 mM *N*-acetylcysteine (NAC) for 1 h and then treated with the APAM (25%) for 2 h, and mitochondria, CL, and nuclei were stained with MTR, NAO, and Hoe, respectively. Bar = 400 μm.

ROS can quickly attack lipids in the membranes, leading to lipid peroxides (LPOs) via peroxy radicals. Therefore, we speculated that the APAM might increase mitochondrial LPO (mLPO). To test this hypothesis, we analyzed the occurrence of LPOs using an LPO-reactive dye L248. Significant L248 puncta were distributed diffusely throughout the cytoplasm in untreated cells possessing healthy nuclei (Figure 6A, upper panels). Notably, more concentrated L248 puncta were seen at the perinuclear sites in untreated cells containing smaller nuclei (indicated by yellow arrows). However, all L248 puncta were not colocalized with mitochondria in the cells. Alternatively, following the APAM treatment, L248 puncta became a cluster at the perinuclear sites, colocalizing with mitochondria (Figure 6A, lower panels). Since Cardiolipin (CL) is the major phospholipid found in the mitochondrial membrane and involved in cell death [38,39,40,41], the APAM might increase its oxidation, leading to production of the oxidized form of CL, CLOOH. The fluorescent dye 10-*N*-nonyl acridine orange (NAO) can bind to CL but not CLOOH [37]. Therefore, NAO staining enables us to judge the oxidative state of CL. We analyzed the mitochondrial NAO staining following the APAM treatment. Substantial NAO signals that entirely colocalized with MTR signals were seen in untreated cells (Figure 6B, top panels). The APAM treatment resulted in marked concurrent decreases in MTR and NAO signals, indicating mitochondrial depolarization and CL oxidation (Figure 6B, second panels). Conversely, Ferrostatin-1 (FS-1), a peroxy radical scavenger [42,43], significantly increased NAO signals compared with untreated cells (Figure 6B, third panels). Moreover, FS-1 prevented the decrease in NAO signals caused by APAM treatment (Figure 6B, fourth panels). NAC treatment also inhibited the decrease in NAO signals while minimally affecting their intensity by itself (Figure 6B, fifth and bottom panels). These results indicate that the APAM can induce CL oxidation through oxidative stress via ROO•. Together, these data demonstrate that MPMC involves mLPO accumulation and CL oxidation.

### 2.7. APAM Shows Similar Biological Effects in OC Cells

**Figure 7:**
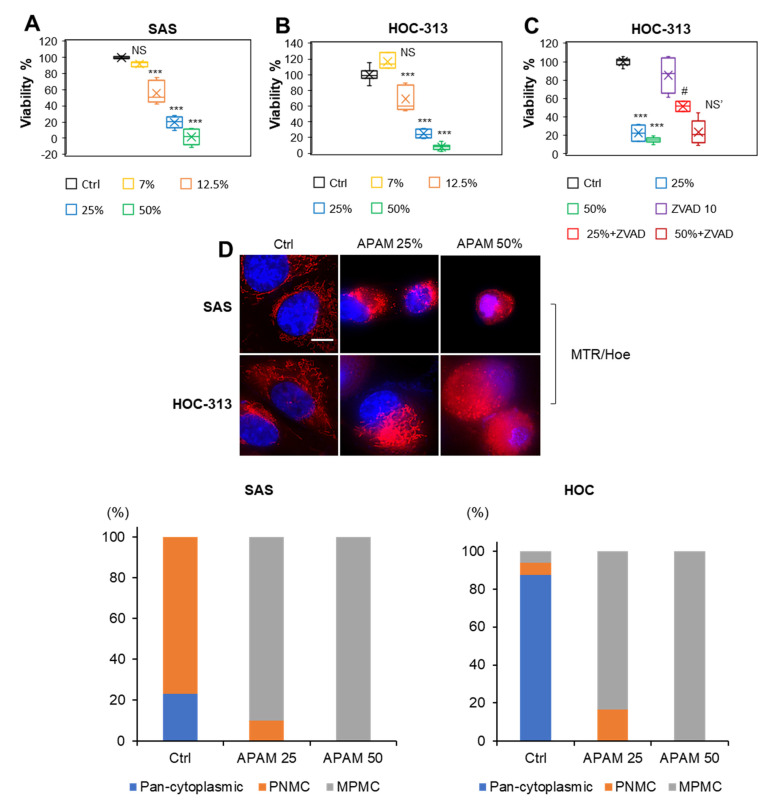

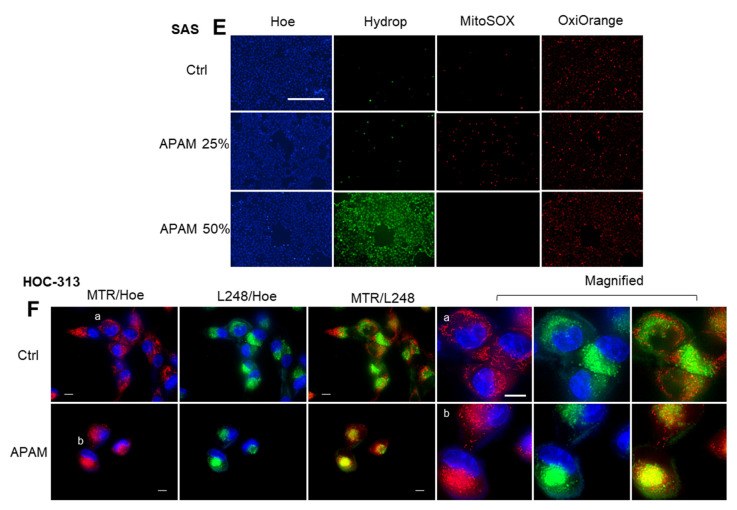
APAM induces cell death, ROS generation, LPO accumulation, and MPMC in OC. (**A**–**C**). SAS (**A**) and HOC-313 (**B**) cells (4 × 10^3^ cells) were treated with the indicated concentrations of the APAM for 72 h and analyzed for viability using a WST-8 cell growth assay. (**C**). HOC-313 cells were pretreated with 10 μM Z-VAD-FMK (ZVAD) for 1 h and then treated with the APAM (25, 50%). Data are the mean ± SD (*n* = 7). Data were analyzed by one-way analysis of variance followed by Tukey’s post hoc test. *** *p* < 0.001; NS, not significant vs. control treated with vehicle. # *p* < 0.05; NS’, not significant vs. APAM alone. (**D**). After treating SAS (upper) and HOC-313 cells (lower) with the APAM (25, 50%) for 18 h, mitochondria and nuclei were stained with MTR and Hoechst 33342 (Hoe), respectively. MTR/Hoe merged images were obtained and analyzed as described in the legend of Figure 4. Bar = 10 μm. For each experimental group, mitochondria showing the three distributions were counted as described in the legend of Figure 4. (**E**). APAM induces H_2_O_2_, superoxide, and hydroxyl radical production. SAS cells (1.5 × 10^4^) were treated with the APAM (25, 50%) for 2 h and then stained with 5 μM MitoSOX, 1 μM Hydrop, or 1 μM OxiOrange. The nuclei were counterstained with Hoe. Bar = 400 μm. (**F**). HOC-313 cells were treated with the APAM (50%) for 2 h, and mitochondria, LPO, and nuclei were stained with MTR, L248, and Hoe, respectively. The magnified images (a,b) show the magnification of a,b in the merged images. Bar = 10 μm.

Next, we sought to determine whether the APAM’s effects were specific for OS or general in different cancers. Therefore, we examined whether the APAM was also effective against OC because they belong to a histologically distinct carcinoma from epithelial tissues. Results showed that the APAM decreased the viability of SAS and HOC-313 cells in a dose-dependent manner (Figure 7A,B). Moreover, Z-VAD-FMK significantly inhibited the effect of the APAM (25%), but not the APAM (50%) (Figure 7C), indicating the involvement in apoptosis and nonapoptotic cell death. The APAM also induced MPMC in the cells dose-dependently (Figure 7D). The APAM also increased the mitochondrial superoxide, hydroxyl radical, and H_2_O_2_ in the cells, as shown by elevated MitoSOX, OxiOrange, and Hydrop signals, respectively (Figure 7E). Additionally, live-cell imaging analysis using L248 revealed a substantial increase in LPO in the mitochondrial cluster in the cells (Figure 7F). Together, these results indicate that the APAM induces cell death and MPMC in OC through possibly similar mechanisms.

### 2.8. APAM Induces Minimal Cell Death, Mitochondrial Oxidative Stress, and MPMC in Noncancerous Cells

**Figure 8:**
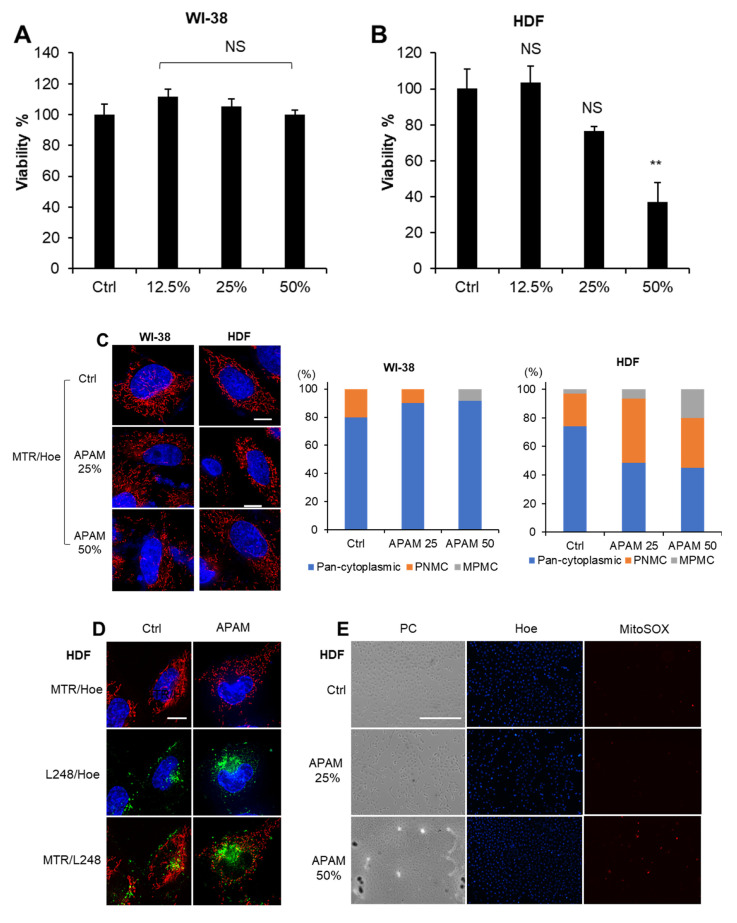

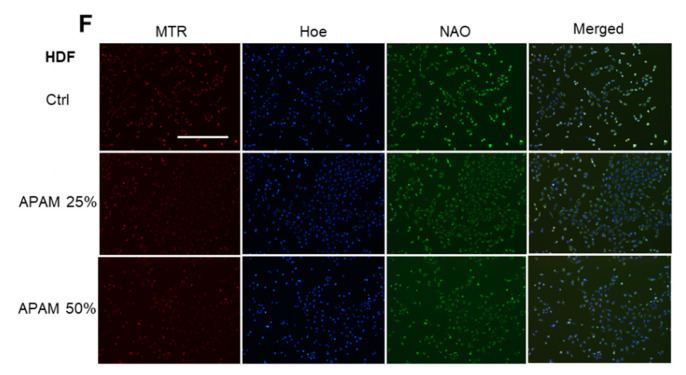
APAM causes minimal cell death, ROS generation, LPO accumulation, CL oxidation, and MPMC in noncancerous cells. (**A**,**B**). WI-38 (**A**) and HDF cells (4 × 10^3^ cells) (**B**) were treated with the indicated concentrations of APAM for 72 h and analyzed for viability using a WST-8 cell growth assay. Data are the mean ± SD (*n* = 3). Data were analyzed by one-way analysis of variance followed by Tukey’s post hoc test. ** *p* < 0.01; NS, not significant vs. control treated with vehicle. (**C**). After treating WI-38 and HDF cells (1.5 × 10^4^) with the APAM (25, 50%) for 18 h, mitochondria and nuclei were stained with MTR and Hoe, respectively. Images were obtained and analyzed as described in the legend of Figure 4. Bar = 10 μm. For each experimental group, mitochondria showing the three distributions were counted as described in the legend of Figure 4. (**D**). HDF cells were treated with the APAM (50%) for 2 h, and mitochondria, LPO, and nuclei were stained with MTR, L248, and Hoe, respectively. Images were taken as described above. Bar = 10 μm. (**E**). HDF cells were treated with the APAM (50%) for 2 h and then stained with 5 μM MitoSOX and Hoechst 33342 (Hoe). PC, phase contrast. Bar = 400 μm. (**F**). HDF cells were treated with the APAM (25, 50%) for 2 h, and mitochondria, CL, and nuclei were stained with MTR, NAO, and Hoe, respectively. Bar = 400 μm.

The APAM showed a much smaller cytotoxic effect on noncancerous cells, such as WI-38 fibroblasts and HDF. The APAM up to 50% had minimal cytotoxicity against the former, while the APAM up to 25% had no significant cytotoxicity in the latter (Figure 8A,B). Also, minimal cell and nuclear damages occurred following the APAM treatment. The APAM substantially increased mitochondrial fragmentation but minimally evoked MPMC in these cells (Figure 8C). Moreover, the APAM induced minimal LPO, superoxide, and CL oxidation increases in the mitochondria (Figure 8D–F). These results indicate that, such as MPMC, mitochondrial oxidative stress occurs in a tumor-specific manner.

## 3. Discussion

In the present study, we investigated the antitumor activity of the APAM and its possible impact on the mitochondrial network and subcellular positioning. The APAM had potent antitumor activity against OS (Figure 1) and OC (Figure 7) in vitro. In contrast, the APAM had minimal cytotoxicity against human dermal and lung fibroblasts (Figure 8), indicating that it acts in a tumor-specific manner. In support of these results obtained in vitro, the APAM significantly suppressed the growth of allograft or xenograft OS tumors with minimal adverse effects (Figure 1). These observations indicate that the APAM has a tumor-specific cytotoxic activity, similar to other various PAM prepared with different gases or culture media [11,12,13,14,15,16]. It is essential to know the mechanisms underlying antitumor action. The APAM could activate apoptosis and nonapoptotic cell death depending on the cell line (Figure 2). Notably, the antitumor activity of the APAM (25%) was substantially inhibited by Z-VAD-FMK, while that of the APAM (50%) was minimally affected by it (Figure 2 and Figure 7). These observations indicate that the APAM can switch cell death modality from apoptosis to nonapoptotic cell death, depending on the concentration. These properties are similar to those of H_2_O_2_ [44] and oxidative phosphorylation (OXPHOS) inhibitors [45]. Both H_2_O_2_ and OXPHOS inhibitors such as FCCP and antimycin A can activate apoptosis and nonapoptotic cell death, depending on the concentration and processing time. Specifically, cell death during the initial 24 h was sensitive to Z-VAD-FMK, while cell death observed during the subsequent 48 h was insensitive [45]. Highly proliferating cancer cells can rapidly consume nutrients in a medium, resulting in an energy-deficient status during prolonged processing. Accordingly, they could be more prone to necrotic cell death than apoptosis. Similar to the APAM, H_2_O_2_ and OXPHOS inhibitors generate mitochondrial superoxide and have antitumor activities through oxidative stress [44,45]. Thus, mitochondrial oxidative stress may lead to different cell death pathways depending on the intensity and duration. Moderate and short stress can activate apoptosis, while intense and persistent stress may be required for nonapoptotic cell death in apoptosis-resistant cells. Ferroptosis, or iron-mediated necrotic cell death, has emerged as another cell death modality activated in various cancers by anticancer compounds [42,43,46]. However, given that there is no mitochondrial requirement for ferroptosis [46], APAM-induced cell death seems different from ferroptosis. The roles of autophagy and ferroptosis in APAM-induced cell death are currently under investigation in our laboratory.

It has been long known that mitochondria are highly plastic, dynamic, and heterogenous organelles. Their size and shape (network shape) vary depending on tissues, cells, and experimental conditions. However, much attention has recently been paid to the biological significance of their heterogeneous properties. An emerging view is that changes in mitochondrial network and location are not merely passive events but active events coupling with cellular functions and survival. Concerted mitochondrial dynamics and subcellular positioning are essential for cell function, survival, and specific cancer onset and progression [24,25]. Mitochondria can distribute throughout the cytoplasm (pan-cytoplasmic), subplasmalemmal, or perinuclear. Precise distributions are essential for energy supply [18,19], the plasma membrane Ca^2+^ channel activity [47], Ca^2+^ signaling [48,49,50], and hypoxic state and signaling [26,27,28,29]. Moreover, several reports demonstrate a closed relationship between mitochondrial motility and the mitochondria-ER tethering [51], Ca^2+^ transport [52], and redox state [53,54]. Our previous works show that mitochondrial fragmentation followed by clustering is essential for apoptotic or nonapoptotic cell death caused by the TRAIL, HePAM, and PAI [12,13,17,32,33]. All these substances modulate the mitochondrial network in tumor cells specifically. HePAM activates both Drp1-dependent and -independent mitochondrial fragmentation, and exogenously added H_2_O_2_ mimics the former mechanism [12]. The present study revealed that the APAM also evoked mitochondrial fragmentation and clustering in a tumor-specific manner. The APAM and HePAM [12,13] are chemically and biologically similar. They commonly have H_2_O_2_, which increases mitochondrial superoxide, thereby inducing mitochondrial depolarization. Notably, both HePAM and H_2_O_2_ [12,13] and the APAM (Figure 8) minimally increased mitochondrial superoxide in normal cells. Additionally, HePAM and H_2_O_2_ can activate Drp1 phosphorylation at Ser 616, the driving force to mitochondrial fission in tumor cells but not normal cells [12,13]. Despite these facts, mitochondrial fragmentation caused by HePAM is augmented but not suppressed by the Drp1 inhibitor Mdivi-1 or Drp1 gene silencing [12]. An intriguing possibility is that mitochondrial fragmentation is triggered by the Drp1-dependent fission pathway via mitochondrial oxidative stress and exacerbated by another Drp1-independent pathway that might play an additional role. Further examination of this scenario is underway.

**Figure 9:**
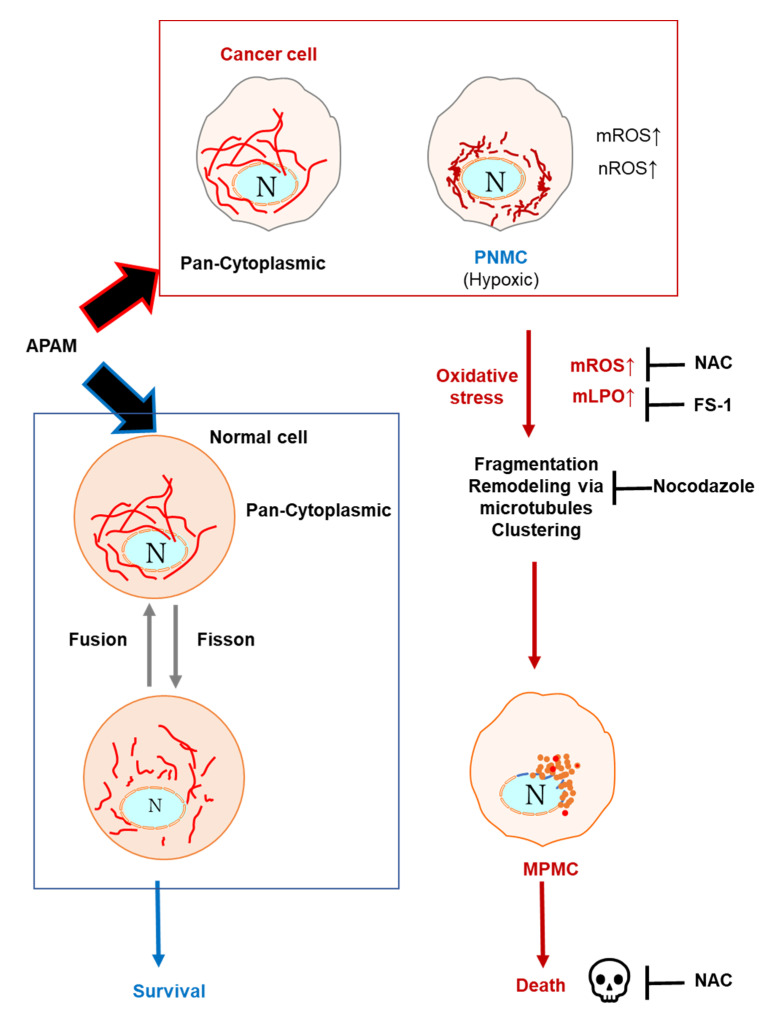
The working model in this study. Cancer cells are adapting to hypoxic microenvironments due to PNMC. Mitochondria in PNMC can generate ROS (mROS), which increases nuclear ROS (nROS), oxidation-mediated HIF-1α stabilization, and downstream hypoxia signaling. Consequently, the mitochondria in cancer cells exhibit pan-cytoplasmic or PNMC distributions, depending on their hypoxic conditions. The APAM induces massive mROS and activates a nonphysiological mitochondrial fragmentation pathway. The fragmented mitochondria will move along with microtubules and form clusters at one side of the perinuclear sites (MPMC), leading to cell death. On the other hand, normal cells possess pan-cytoplasmic mitochondria, and the APAM increases mitochondrial oxidative stress only modestly. It predominantly causes the physiological Drp1-dependent mitochondrial fission, reversible through the fission-fusion dynamics, leading to cell survival.

Moreover, we discovered that the mitochondrial network collapse eventually led to a unique mitochondrial subcellular positioning, MPMC. This event occurs in all tumor cell lines tested (Figure 4 and Figure 7) but not lung and dermal fibroblasts (Figure 8). It is critical to determine whether MPMC is a cause or a consequence of death. To address this question, we compared the kinetics of MPMC and cell death. Results indicated that MPMC was initiated as rapidly as within hours after the APAM treatment. At that timepoint, minimal cell and membrane damages occur. Similar to cell death, MPMC was prevented by NAC (Figure 5). Thus, MPMC is unlikely to be merely a consequence of cell death. Instead, it may be a prodeath event where ROS play a role. At present, the precise mechanisms underlying MPMC remain unclear. However, the simultaneous occurrence of mROS (Figure 3 and Figure 7), mLPO accumulation, and CL oxidation as well as prevention by FS-1 (Figure 6 and Figure 7) indicate the critical role of mLPO accumulation. Moreover, concomitant translocation of the mitochondrial and tubulin networks, and their prevention by NC (Figure 5), strongly suggest the involvement of mitochondrial motility through the action of microtubule-associated motor proteins. Together, our data coincide with the working model shown in Figure 9. The APAM increases mROS, including superoxide, H_2_O_2_, and hydroxyl radical, promoting nonphysiological mitochondrial fragmentation. Then, the fragmented mitochondria move along with microtubules and gather at one side of the perinuclear sites. This working model raises several interesting questions, including how MPMC leads to cell death, why it occurs tumor-selectively, and why mitochondria gather in unipolar perinuclear regions. Since MPMC is pronounced explicitly in cells possessing shrank and decomposed nuclei, it might play a role in nuclear damages. Given the critical role of PNMC in hypoxia, MPMC might also compromise hypoxic status in cancer cells. PNMC has been shown to contribute to the stabilization of HIF-1α. Mitochondria in PNMC can generate ROS (mROS), which in turn increases nuclear ROS (nROS), leading to oxidation-mediated HIF-1α stabilization and downstream hypoxia signaling [26,27,28,29]. In addition, activated metabolism and genetic instability under the control of oncogenic transformations cause increased ROS generation and decreased antioxidant systems in cancer cells. Thus, cancer cells may be exposed to higher oxidative stress than normal cells. Therefore, the APAM may preferentially make tumor cells exceed the allowed mitochondrial oxidative stress range and undergo MPMC. Finally, we are currently attempting to discern the biochemical characteristics of the perinuclear sites in which mitochondria make clusters. H_2_O_2_ is long-lived species found in the APAM (this study) and has been shown to play a primary role in various types of PAM [11,12,13,14]. Moreover, the oxidant can mimic the antitumor activity, the tumor-selective activity, and the capacity to induce mitochondrial fragmentation of HePAM [12]. Therefore, the oxidant is the most likely candidate for the primary mediator in the APAM’s action. However, preliminary experiments showed that exogenously added H_2_O_2_ could not fully mimic the APAM’s capacity to cause MPMC. These observations suggest the involvement of another oxidant. Recent evidence indicates that RNS, including peroxynitrite, are also involved in PAM cytotoxicity [18,19,20,21]. Therefore, it is possible that H_2_O_2_ and RNS cooperatively mediate MPMC and cell death. Further investigation to test this possibility is ongoing.

In summary, the present study shows that a tumor-targeting agent, the APAM, can switch the mitochondrial positioning from PNMC to MPMC in OS and OC cells but not fibroblasts. Cancer cells seem to be more prone than normal cells to the switch due to their intense ambient oxidative stress. Thus, MPMC might serve as a new promising target for exerting tumor-specific cytotoxicity.

## 4. Materials and Methods

### 4.1. Materials

All chemicals were purchased from Sigma Aldrich (St. Louis, MO, USA) unless otherwise specified. The pan-caspase inhibitor Z-VAD-FMK was purchased from Merck Millipore (Darmstadt, Germany). All insoluble reagents were dissolved in dimethyl sulfoxide (DMSO) and diluted with high glucose-containing Dulbecco’s modified Eagle’s medium (DMEM), supplemented with 10% fetal bovine serum (FBS) or Hank’s balanced salt solution (HBSS; pH 7.4, Nissui Pharmaceutical Co., Ltd., Tokyo, Japan) (final DMSO concentration, <0.1%) before use.

### 4.2. Cell Culture

The human OC cell line, SAS (JCRB0260), was obtained from the Japanese Collection of Research Bioresources (JCRB) Cell Bank of National Institutes of Biomedical Innovation, Health, and Nutrition (Osaka, Japan). Another human OC cell line, HOC313, was kindly provided by the Department of Oral and Maxillofacial Surgery, Graduate School of Medical Science, Kanazawa University (Kanazawa, Japan). Human fetal lung fibroblasts, WI-38 (JCRB9017), were obtained from JCRB. Human dermal fibroblasts (HDFs) from the facial dermis were obtained from Cell Applications (San Diego, CA, USA). Human osteosarcoma HOS (RCB0992) and 143B (RCB0701), as well as the murine osteosarcoma LM8 (RCB1450), cells were purchased from Riken Cell Bank (Tsukuba, Japan). The cells were maintained in 10% FBS (GIBCO^®^, Life Technologies) containing DMEM (GIBCO^®^, Life Technologies, Carlsbad, CA, USA) (FBS/DMEM) supplemented with 100 U/mL penicillin and 100 μg/mL streptomycin at 37 °C in a humidified atmosphere with 5% CO_2_.

### 4.3. APAM Preparation

CAP was generated from the ambient air using a Piezobrush™ PZ2 model plasma jet (relyon, Germany) equipped with a piezo element. The typical experimental conditions are a frequency of >50 kHz, a voltage of >20 kV, and an electron density of 10^14~16^/cm. The APAM (1 mL) was made by irradiating plasma from above at a distance of 20 mm to 1 mL of DMEM without phenol red for 1 min. The original APAM was diluted to a final concentration of 6.3–50% with 10% FBS/DMEM (for cell experiments) or HBSS (for biochemical experiments) and was indicated as the APAM (6.3–50%).

### 4.4. Cell Viability Assay

Cell viability was measured by the WST-8 assay using Cell Counting Reagent SF (Nacalai Tesque, Inc., Kyoto, Japan) or Cell Counting Kit-8 (Dojindo Molecular Technologies, Inc., Kumamoto, Japan) according to the manufacturer’s instructions. These methods are colorimetric assays based on the formation of a water-soluble formazan product (WST-8). In short, cells were seeded at a density of 3 or 4 × 10^3^ cells/well in 96-well plates (Corning Incorporated, Corning, NY, USA). They were cultured with agents to be tested for 72 h at 37 °C before adding 10 μL cell counting reagent and further incubation for 2 h. Absorbances at 450 nm were measured using a Nivo (PerkinElmer Japan Co., Ltd., Yokohama, Japan) or SH-1100R (Lab) microplate reader (Corona Electric Co., Ltd., Ibaraki, Japan).

### 4.5. Animals and In Vivo Antitumor Activity

All animal experiments were conducted according to the Guidelines for Proper Conduct of Animal Experiments, Science Council of Japan. Protocols were approved by the Animal Care and Use Committee (No.17-11), University of Yamanashi. Male C3H/HeJJcl mice and BALB/cAJcl-nu/nu mice were purchased from CLEA Japan, Inc. (Tokyo, Japan). The mice were housed at 22–24 °C under a 12-h light/dark cycle and were fed standard mouse chow. Water was available ad libitum. The ability of the APAM to reduce tumor growth in vivo was evaluated using allograft transplants of LM8 cells in mice. Male C3H/HeJJcl or BALB/cAJcl-nu/nu mice (8 weeks of age) were administered general anesthesia with isoflurane (ISOFLU; Abbott Laboratories, North Chicago, IL, USA) and oxygen. LM8 cells (1 × 10^6^ cells/mouse) in 100 µL DMEM were injected subcutaneously into the backs of the CH3 mice on day 0. Alternatively, 143B cells (1 × 10^6^ cells/mouse) in 100 µL of DMEM were injected intramedullary into the right tibia of the BALB/cAJcl-nu/nu mice on day 0. On day seven post-transplant, 200 μL of the APAM or vehicle were administered intravenously to six mice in each group every other day. The mice were weighed, and the primary tumors were measured weekly. A schematic diagram of the experiment is shown in Supplementary Figure S1.

### 4.6. Cell Death Assay

Cells were cultured in 6-well plates for 24 h and then exposed to the APAM (50%) for 24 h. The cells were retrieved using Versene (GIBCO^®^, Life Technologies) and incubated with APC-conjugated Annexin V and 7-AAD (BD Biosciences) for 15 min to evaluate apoptotic cell death. Data were collected using a FACSCelesta™ flow cytometer (BD Biosciences, Franklin Lakes, NJ, USA). Data obtained were analyzed by CellQuest Pro (Becton Dickinson Biosciences) and FlowJo software (TreeStar, Ashland, OR, USA). Experiments were performed in triplicate. Annexin V-positive cells were defined as apoptotic cells.

### 4.7. Western Blotting Analysis

LM8 and 143B cells were collected, and cell lysates were prepared using a CelLytic MT cell lysis reagent (Sigma-Aldrich, St. Louis, MO, USA) according to the manufacturer’s instructions. Western blotting analysis was performed as previously reported [17]. In short, equal amounts of protein from each sample were analyzed by immunoblotting with primary antibodies against cleaved caspase-3 (Asp175) (5A1E) (#9664, 1:1000), caspase-3 (#9662, 1:1000), LC3-A/B (D3U4C) (#12741, 1:1000), LC3-B (D11) (#3368, 1:1000), and GAPDH (D16H11) (#5174, 1:1000) (Cell Signaling Technology, Danvers, MA, USA). Images were captured using a LAS-4000 camera system from Fujifilm (Tokyo, Japan) and quantified using ImageJ 1.52a (Wayne Rasband, National Institutes of Health, Bethesda, MD, USA).

### 4.8. Mitochondrial Network and Positioning Assays

The mitochondrial network in live cells was analyzed as previously described [17]. In short, cells in FBS/DMEM (3 × 10^4^/well) adherent on 8-well chambered coverslips were treated with the agents to be tested for 24 h at 37 °C in a 5% CO_2_ incubator. After removing the medium by aspiration, the cells were washed with fresh FBS/DMEM and stained with 20 nM MitoTracker^TM^ Red CMXRos (MTR) for 1 h at 37 °C in the CO_2_ incubator. In positioning experiments, the nuclei were counterstained with 1 mg/mL of Hoechst 33342. The cells were then washed and immersed in FluoroBrite^TM^ DMEM (Thermo Fisher Scientific, Waltham, MA, USA). Images were obtained using a BZ X-710 Digital Biological Microscope (Keyence, Osaka, Japan) equipped with a 100×, 1.40 n.a. UPlanSApo Super-Apochromat coverslip-corrected oil objective (Olympus, Tokyo, Japan) and analyzed using BZ-H3A application software (Keyence). The occupied mitochondrial area was measured in three photos per sample using the BZ-H3M application, as shown in Supplementary Figure S3. For each experimental group, the mitochondria (≤40) in at least two different pictures were counted for three different subcellular distributions (pan-cytoplasmic, PNMC, and MPMC), and the percentage was shown (*n* = 2–4).

### 4.9. Autophagy Assay

Cells were cultured in 6-well plates for 18 h and then exposed to the APAM (50%) for 24 h. Cells were stained and analyzed using DAL Green (Dojindo, Kumamoto, Japan) according to the manufacturer’s instructions. Data were collected using a FACSCelesta™ flow cytometer (BD Biosciences, Franklin Lakes, NJ, USA) and analyzed using FlowJo software (TreeStar Inc., Palo Alto, CA, USA). Experiments were performed in triplicate. In mitophagy experiments, mitochondria and autophagosomes were stained with MTR and the specific dye Cyto-ID^®^ (Enzo Life Sciences, Farmingdale, NY, USA), respectively. Mitophagy was judged from colocalization between mitochondria and the Cyto-ID signals.

### 4.10. Quantitation of H_2_O_2_ in APAM

The concentration of H_2_O_2_ in the APAM was measured using the Amplex Red Hydrogen Peroxide/Peroxidase Assay Kit (Thermo Scientific, Rockford, IL, USA) according to the manufacturer’s protocols as previously described [17]. In short, samples were diluted appropriately and placed on a 96-well plate (50 μL/well). Then, 50 μL of a working solution of 100 μM of Amplex Red reagent and 0.2 U/mL of horseradish peroxidase were added to the well and incubated at room temperature for 30 min. The absorbance at 570 nm was measured using a Nivo 3F Multimode Plate Reader (PerkinElmer Japan Co., Ltd., Yokohama, Japan). The concentrations of H_2_O_2_ were calculated using a standard curve made using the authentic H_2_O_2_ from the kit.

### 4.11. Measurements of Intracellular ROS Generation

Cells (1.5 × 10^4^/well) in FBS/DMEM were cultured on a 35-mm poly-Lysine-coated glass-bottom dish (Matsunami Glass, Tokyo, Japan) and treated with the agents to be tested for 2 h at 37 °C at a CO_2_ incubator. After removing the medium by aspiration, the cells were placed in FluoBrite^TM^ DMEM and stained with 5 μM MitoSOX^TM^ Red (MitoSOX, Thermo Fisher Scientific), 1 μM OxiOrange^TM^, or 1 μM Hydrop^TM^ (Goryo Chemicals, Sapporo, Japan) for 20 min. Images were obtained with EVOS FL Cell Imaging System (Thermo Fisher Scientific) equipped with a 10 ×objective and analyzed using the freely available NIH ImageJ software (NIH, Bethesda, MD, USA) as previously described [30].

### 4.12. LPO and CL Oxidation Analyses

Cells (3 × 10^4^/well) were cultured and treated as described above and stained with 20 nM MitoTracker Red CMXRos, 1 μM L248 (Liperfluo, Dojindo), or 100 nM 10-*N*-Nonyl acridine orange (NAO, Sigma-Aldrich) for 20 min. Images were obtained with BZ X-710 Digital Biological Microscope (Keyence) or EVOS FL Cell Imaging System (Thermo Fisher Scientific). They were analyzed using BZ-H3A/H3M application (Keyence) or NIH ImageJ software (NIH) as described above. LPO accumulation in mitochondria was judged by colocalization between MTR and L248 signals in merged images.

### 4.13. Statistical Analysis

Data are presented as mean ± standard deviation (SD) and were analyzed by one-way analysis of variance followed by Tukey’s post hoc test using an add-in software with Excel 2016 for Windows (SSRI, Tokyo, Japan). For some experiments, significance was determined using Student’s *t*-test after an f-test. *p* < 0.05 was considered statistically significant.

## Supplementary Materials

Supplementary Materials can be found at https://www.mdpi.com/article/10.3390/ijms23031124/s1.
