# Supplementary material for: Air Plasma-Activated Medium Evokes a Death-Associated Perinuclear Mitochondrial Clustering
Source: Int J Mol Sci. 2022 Jan 20;23(3):1124. doi: 10.3390/ijms23031124 (PMC8835529; doi:10.3390/ijms23031124)
Supplement: Supplementary file 1 [file ijms-23-01124-s001.zip › Supplementary Materials.pptx]

## Slide 1
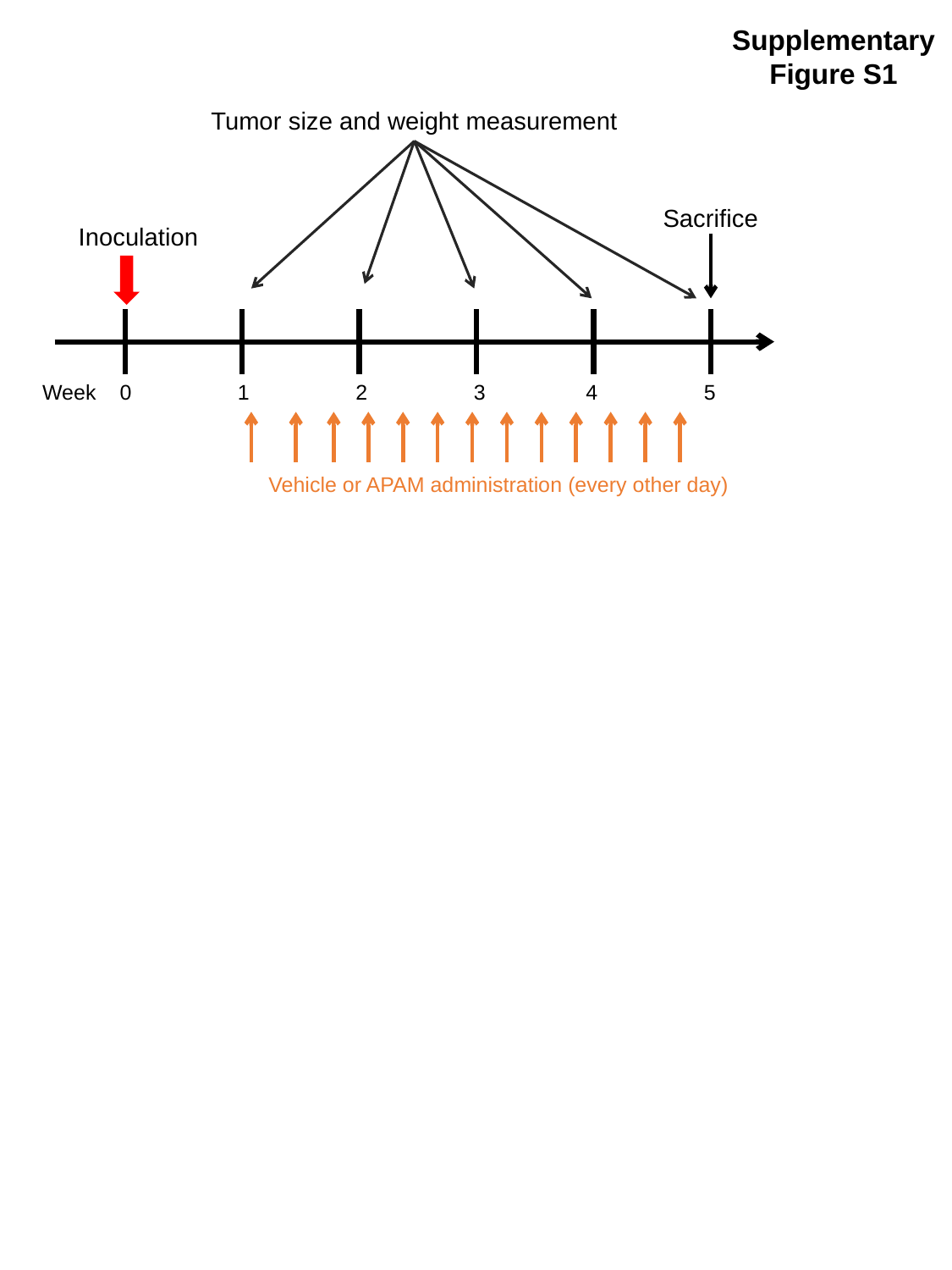

Supplementary
Figure S1
Tumor size and weight measurement
Sacrifice
Inoculation
Week 0 1 2 3 4 5
Vehicle or APAM administration (every other day)

## Slide 2
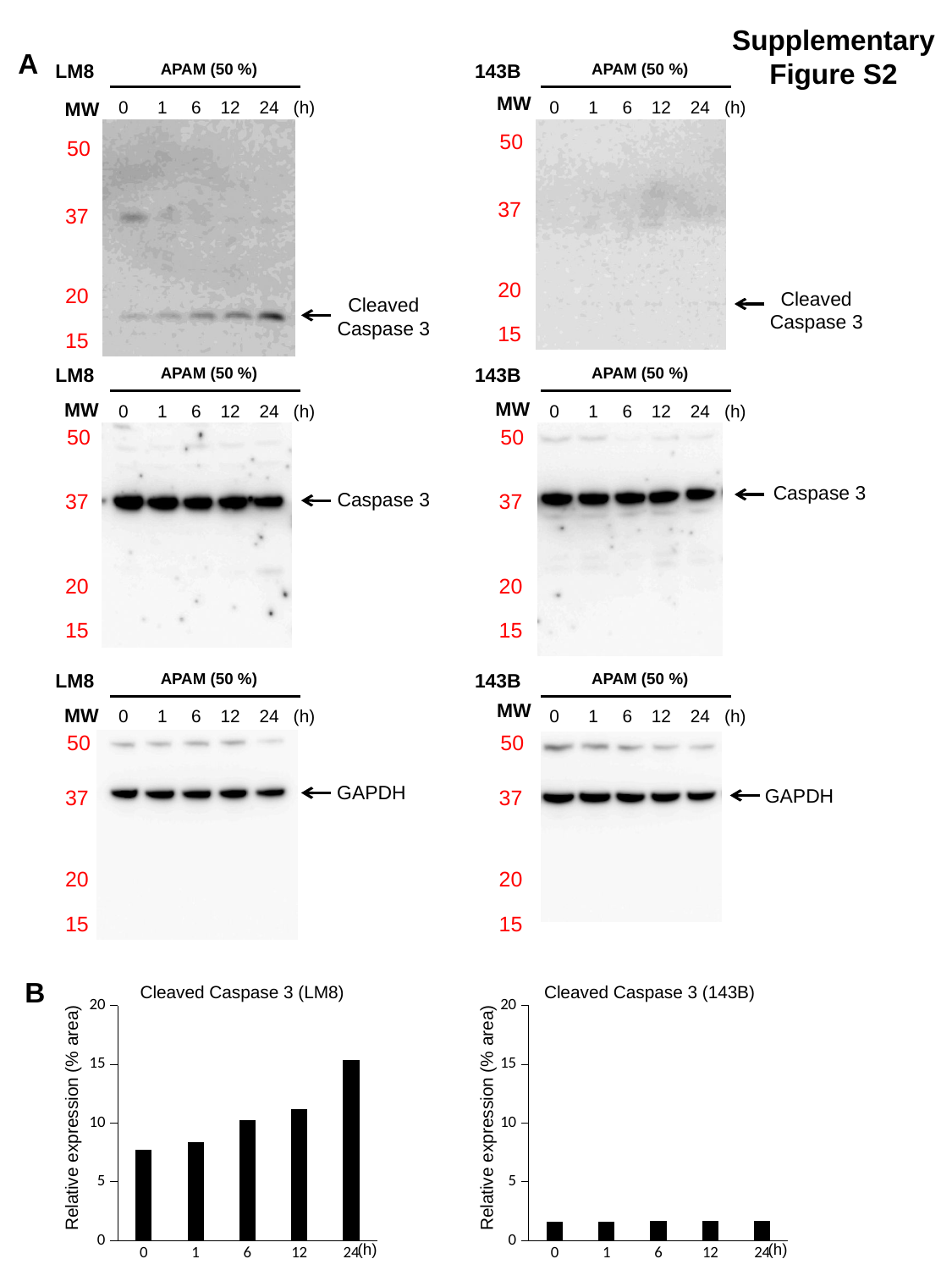

Supplementary
Figure S2
A
LM8
143B
APAM (50 %)
APAM (50 %)
0 1 6 12 24 (h)
0 1 6 12 24 (h)
MW
MW
50
50
37
37
20
20
Cleaved Caspase 3
Cleaved Caspase 3
15
15
LM8
143B
APAM (50 %)
APAM (50 %)
0 1 6 12 24 (h)
0 1 6 12 24 (h)
MW
MW
50
50
Caspase 3
Caspase 3
37
37
20
20
15
15
LM8
143B
APAM (50 %)
APAM (50 %)
0 1 6 12 24 (h)
0 1 6 12 24 (h)
MW
MW
50
50
GAPDH
GAPDH
37
37
20
20
15
15
B
Cleaved Caspase 3 (LM8)
Cleaved Caspase 3 (143B)
### Chart
| Category | |
|---|---|
| 0 | 7.695969498910675 |
| 1 | 8.371132897603486 |
| 6 | 10.262200435729847 |
| 12 | 11.171023965141611 |
| 24 | 15.396949891067537 |
### Chart
| Category | |
|---|---|
| 0 | 1.611328125 |
| 1 | 1.6176269531249998 |
| 6 | 1.6681640625 |
| 12 | 1.6466796874999998 |
| 24 | 1.64189453125 |Relative expression (% area)
Relative expression (% area)
(h)
(h)

## Slide 3
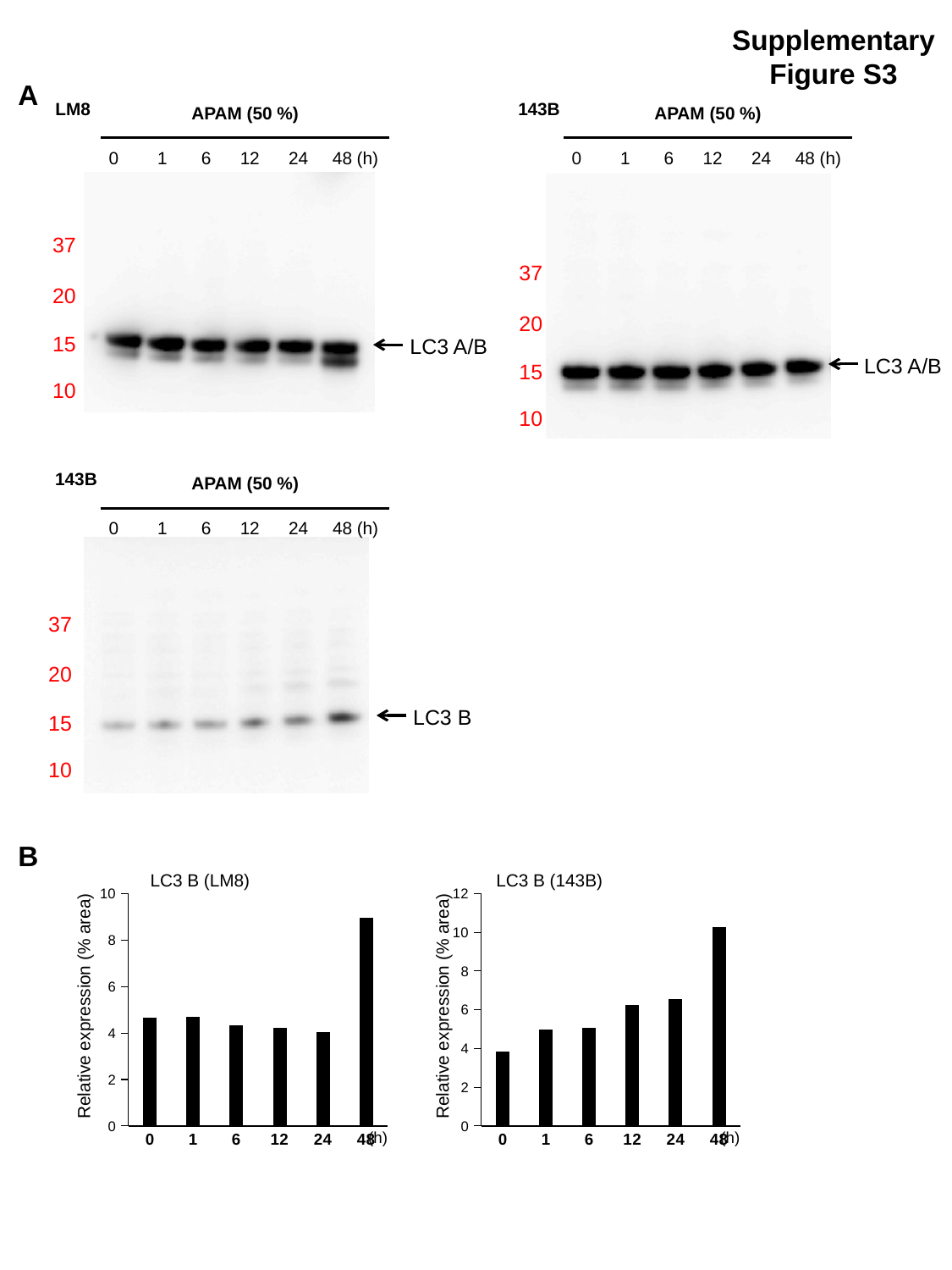

Supplementary
Figure S3
A
LM8
143B
APAM (50 %)
APAM (50 %)
0 1 6 12 24 48 (h)
0 1 6 12 24 48 (h)
37
37
20
20
15
LC3 A/B
LC3 A/B
15
10
10
143B
APAM (50 %)
0 1 6 12 24 48 (h)
37
20
LC3 B
15
10
B
LC3 B (LM8)
LC3 B (143B)
### Chart
| Category | |
|---|---|
| 0 | 4.653479853479854 |
| 1 | 4.684554334554335 |
| 6 | 4.321123321123322 |
| 12 | 4.217094017094017 |
| 24 | 4.0226495726495735 |
| 48 | 8.942612942612941 |
### Chart
| Category | |
|---|---|
| 0 | 3.825223435948362 |
| 1 | 4.9828202581926515 |
| 6 | 5.060377358490566 |
| 12 | 6.247070506454817 |
| 24 | 6.569910625620656 |
| 48 | 10.267825223435949 |Relative expression (% area)
Relative expression (% area)
(h)
(h)

## Slide 4
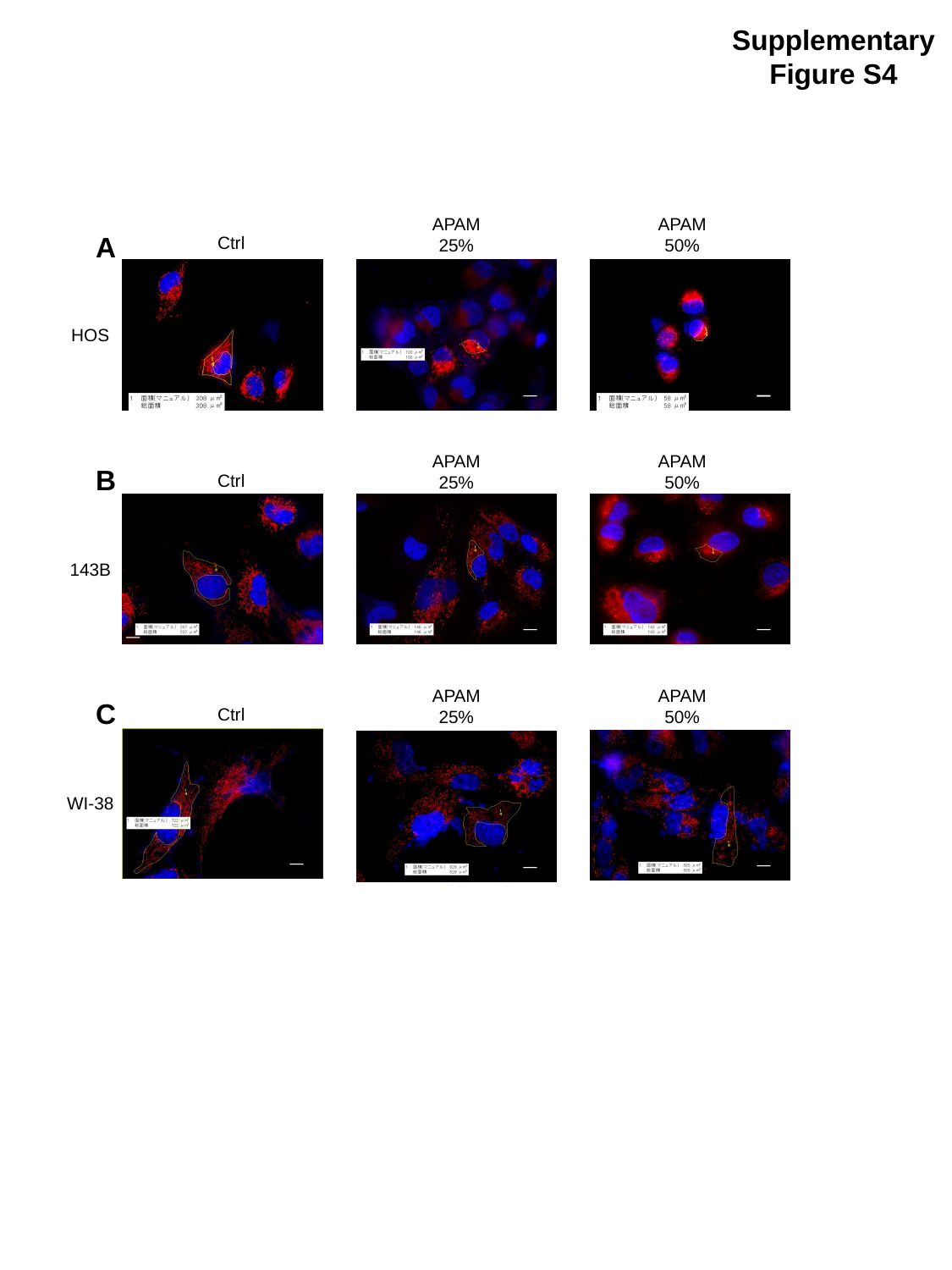

Supplementary
Figure S4
APAM
25%
APAM
50%
A
Ctrl
HOS
APAM
25%
APAM
50%
B
Ctrl
143B
APAM
25%
APAM
50%
C
Ctrl
WI-38

## Slide 5
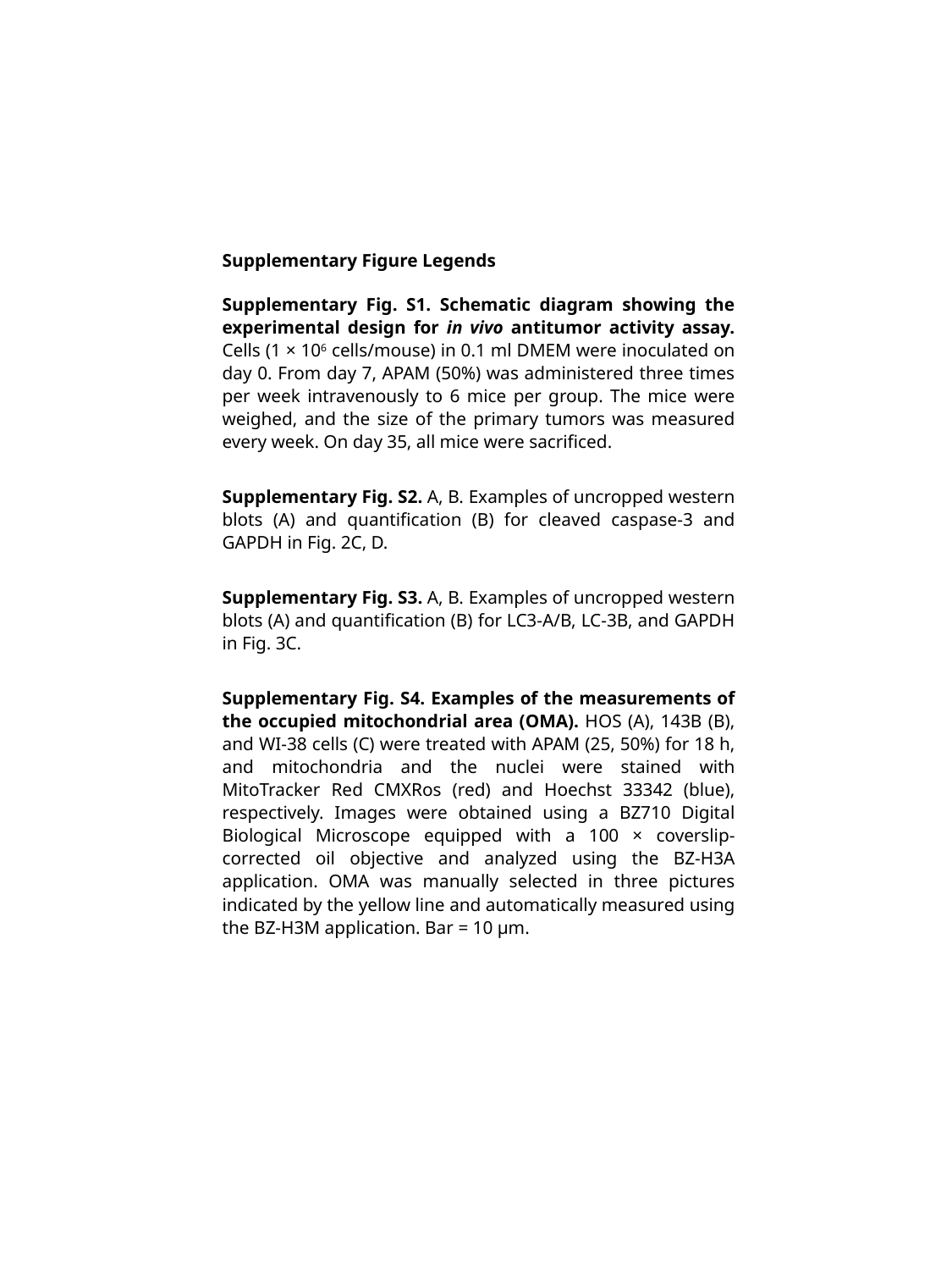

Supplementary Figure Legends
Supplementary Fig. S1. Schematic diagram showing the experimental design for in vivo antitumor activity assay. Cells (1 × 10^6^ cells/mouse) in 0.1 ml DMEM were inoculated on day 0. From day 7, APAM (50%) was administered three times per week intravenously to 6 mice per group. The mice were weighed, and the size of the primary tumors was measured every week. On day 35, all mice were sacrificed.
Supplementary Fig. S2. A, B. Examples of uncropped western blots (A) and quantification (B) for cleaved caspase-3 and GAPDH in Fig. 2C, D.
Supplementary Fig. S3. A, B. Examples of uncropped western blots (A) and quantification (B) for LC3-A/B, LC-3B, and GAPDH in Fig. 3C.
Supplementary Fig. S4. Examples of the measurements of the occupied mitochondrial area (OMA). HOS (A), 143B (B), and WI-38 cells (C) were treated with APAM (25, 50%) for 18 h, and mitochondria and the nuclei were stained with MitoTracker Red CMXRos (red) and Hoechst 33342 (blue), respectively. Images were obtained using a BZ710 Digital Biological Microscope equipped with a 100 × coverslip-corrected oil objective and analyzed using the BZ-H3A application. OMA was manually selected in three pictures indicated by the yellow line and automatically measured using the BZ-H3M application. Bar = 10 μm.
